# Digital Detox

**DOI:** 10.1007/s12599-022-00747-x

**Published:** 2022-02-22

**Authors:** Milad Mirbabaie, Stefan Stieglitz, Julian Marx

**Affiliations:** 1 Faculty of Business Administration and Economics, Paderborn University, Warburger Str. 100, (Q3.128), 33098 Paderborn, Germany; 2 Department of Computer Science and Applied Cognitive Science, Faculty of Engineering, Digital Communication and Transformation, University of Duisburg-Essen, Forsthausweg 2, 47057 Duisburg, Germany

**Keywords:** Digital detox, Technostress, Knowledge work, Information technology

*“We must learn to reawaken and keep ourselves awake, not by mechanical aids, but by an infinite expectation of the dawn, which does not forsake us even in our soundest sleep. I know of no more encouraging fact than the unquestionable ability of man to elevate his life by a conscious endeavour.”* ― Henry David Thoreau, Walden

## Introduction

The increasing use of information technology (IT) has a pervasive impact on society, including the world of work and its boundaries. Individual professionals, and knowledge workers in particular, are exposed to digital devices during the bulk of their working hours (Orlikowski and Scott 2016). In addition, persuasively designed social media and digital entertainment applications occupy the leisure time of an unprecedented number of people. A recent study revealed that 33.1 million Germans use the Internet *“multiple times a day”*, and 11 million even state to use it *“constantly, almost the whole time”* (Statista 2020). Scholarship clearly suggests that this compounded screen time can entail severe consequences to the wellbeing of individuals (Pflügner et al. 2020a). In fact, using IT can lead to technostress, which is defined as “*any negative impact on attitudes, thoughts, behaviors, or body physiology that is caused either directly or indirectly by technology*” (Weil and Rosen 1997, p. 5). Technostress constitutes a pressing social issue, especially with regards to changes in work-life boundaries, potentiated by the COVID-19 pandemic (Thomas et al. 2020). According to a study conducted in 2019, 86 percent of participants claimed that the inability to switch off devices after regular working hours has a negative effect on employee wellbeing (Stewart 2020). The result is a personal feeling of being overwhelmed by communication content and interpersonal online connections, which negatively affects work and private life alike (Gui and Büchi 2019).

To counteract technostress and its negative consequences on individual wellbeing and productivity, the notion of “digital detox” has found its way into popular culture and, more recently, Information Systems (IS) scholarship (Vaghefi et al. 2018; Eichner 2020; Zhou et al. 2020). Digital detox describes a periodic disconnection from IT as well as strategies which help to reduce the engagement with IT (Syvertsen and Enli 2019). Both its conceptualization and empirical analysis, however, have so far remained vague. Early research presents mixed results concerning the effectiveness of digital detox to improve individual wellbeing (Wilcockson et al. 2019; Brown and Kuss 2020; Schmuck 2020). Yet, making a statement about its effectiveness largely depends on the way digital detox is defined in each individual study. Despite this ambiguity, however, the literature commonly stresses the importance of remaining absent from IT for specified periods and calls for more research on this matter. The growing demand for digital detox before, during, and most likely after the COVID-19 pandemic fundamentally questions the way we use IT. Individuals increasingly find themselves yearning for time without the pervasive presence of IT (Fu et al. 2020). Digital detox, we argue, poses a symptom of a serious problem, that is, detrimental effects of IT use on health and work satisfaction. How can IS research help to get to the root of this problem?

**Table:** 

**Digital Detox**
Digital detox describes a periodic disconnection from IT as well as strategies which help to reduce the engagement with IT (adapted from Syvertsen and Enli 2019)

In this article, we demonstrate the rationale behind digital detox and the developments in organizational knowledge work that precipitate the increasing popularity of periodically refraining from the use of IT. Moreover, we propose a first conceptualization of digital detox to guide future research in IS and beyond.

## Technostress in Organizations

IT pervasively affects individuals’ private and professional life (Tarafdar et al. 2019). Work arrangements built around steady IT use have become commonplace, in particular for knowledge workers (Kissmer et al. 2018). The latter are defined as workers whose occupation relies on “*the creation, distribution or application of knowledge*” (Davenport 2005, p. 9) Typical work arrangements allow knowledge workers to connect with people in geographical proximity, across greater distances, or completely remote—independently of time and space (Frick and Marx 2021). The COVID-19 pandemic has impelled a bulk of the workforce to switch to remote work arrangements, and home office respectively (Brynjolfsson et al. 2020). Whereas this development may be expected to be only temporary, leading firms such as Microsoft or Siemens have announced to preserve the ratio of remote work arrangements compared to regular office work beyond the pandemic (Newman 2020). This permanent shift means an empowerment of the knowledge worker in terms of her mobility and autonomy, while dismissing the paradigm of the corporate 9 to 5 job applied to knowledge work (Wang et al. 2020).

The flipside of the coin is that, with increasing IT use due to remote work arrangements, knowledge workers are exposed to a higher risk of technostress (Chandra et al. 2019). This phenomenon refers to stress individuals experience because of their IT use and their inability to cope with it healthily (Riedl et al. 2012; Mahapatra and Pillai 2018; Sarabadani et al. 2020). As employees often have to adapt to new and changing IT implemented by their organization, a number of scholars in IS focus on employee and IT professional related technostress (Chiu 2018; Mahapatra and Pillai 2018; Sarabadani et al. 2020).

### Theoretical Underpinnings of Technostress Literature

Theoretically, the technostress literature heavily builds on the transactional model of stress (Lazarus and Folkman 1984). According to this model, individuals react cognitively to stimuli by assessing the motivational significance of a situation (primary appraisal). This may result in the perception of a situation to be irrelevant, benign-positive, or stressful. Subsequently, according to the model, individuals evaluate the assessment by contemplating possible actions to manage the situation (secondary appraisal). For example, one tries to find ways of alleviating possible harm in case of a stressful situation. The stressors perceived in the situation, in turn, provoke a stress reaction that can be of physiological, emotional, cognitive, or behavioral nature (strain). Finally, the individual may suffer from consequences caused by stress.

So far, IS literature has built on this model in a threefold manner. First, the transactional model has been tailored to technostress in organizations, defining the dimensions job characteristics, technological environment, organizational environment, and social environment, in which stressors can occur. Moreover, consequences of technostress do not only affect individual wellbeing, but may also impair performance, productivity, and IT user satisfaction (Adam et al. 2017). Second, specific techno-stressors have been defined, as shown in Table 1.

**Table 1:** Techno-stressors according to Adam et al. (2017), Tarafdar et al. (2019), and Pflügner et al. (2021)

Techno-stressor	Description
Techno-overload	Technology urging employees to work more and faster
Techno-invasion	Constant availability; blurring work/life boundaries
Techno-complexity	A perceived lack of abilities to meet the demands of IT use
Techno-insecurity	Fearing to lose one’s job to IT or IT-savvy contenders
Techno-uncertainty	Uncertainty about changes in existing or new systems
Techno-unreliability	System malfunctions

Third, technostress research has explored so-called coping strategies to mitigate technostress. With reference to the transactional perspective, these strategies can be problem-focused, e.g., stress-sensitive systems that provide live bio-feedback (Adam et al. 2017), or emotion-focused, e.g., mindfulness exercises (Pflügner et al. 2021). What the technostress literature has in common is that proposed coping strategies set in only after the second appraisal, i.e., after a stressor has been experienced and assessed as such. This constraint opens a new theoretical angle to approach technostress.

### A Digital Detox Perspective on Technostress

**Fig. 1:**
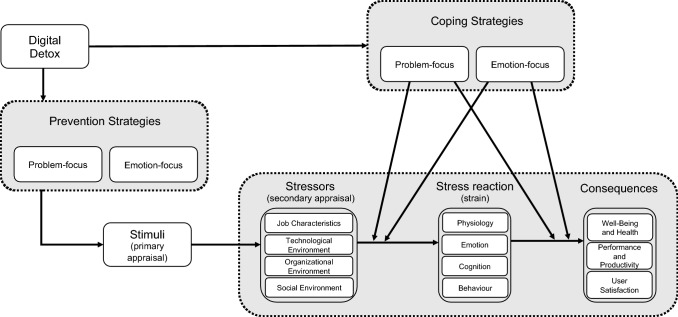
The role of digital detox in the model of organizational technostress (adapted from Adam et al. 2016)

Research on technostress in organizations is guided by the assumption that individual exposure to techno-stressors is determined by job characteristics or technological, organizational, and social environments. Intervention through digital detox, we argue, can also start earlier, i.e., prior to the individual being exposed to the stimuli and techno-stressors, respectively. Figure 1 summarizes this supposed theoretical relationship.

If understood as a periodic disconnection and strategies to reduce the engagement with IT, digital detox has been investigated sporadically in technostress literature in the form of coping strategies. Under the label of ‘behavioral disengagement’, Hauk et al. (2019) describe the phenomenon of an individual “*breaking off any further interaction and withdrawing from the stressful situation*” (p. 22). Interestingly, the authors found this behavior to be counter-productive, with stress levels reducing in the short-term, but then yoyoing back once the individual returns to the unresolved situation. Another study examined coping strategies which specifically answered to techno-invasion and techno-overload in organizations. Here, the authors proposed communication measures for employees and management that might be able to reduce the exposure to IT and its demands (Pflügner et al. 2020b). Strategies specifically aiming at the *reduction of stimuli and successive stressors* in the context of technostress in organizations, however, have not been part of the debate. Instead of changing the independent variable (IT exposure), research has focused on finding appropriate moderating forces (coping strategies) that alter the stress reactions and consequences. Digital detox, in this sense, offers an additional perspective (prevention strategies) that alleviates the predetermined experience of technostress when performing knowledge work.

## The Concept of Digital Detox

Recently, the notion of digital detox has received increasing attention in academia, popular culture, and the self-help industry. The term ‘detox’ itself describes “*a process or period of time in which one abstains from or rids the body of toxic or unhealthy substances*” (Oxford Languages 2021). In medicine, the scientific grounding for detoxification is controversial – it can rather be seen as a consumer buzz word in conjunction with healthcare products (Cohen 2007). In the digital context, however, the effectiveness of detox measures is currently being scrutinized. Extant literature on digital detox goes back to around 2015 (Ugur and Koc 2015) and is dispersed across disciplines such as Psychology (Schmuck 2020), Media and Communication Studies (Syvertsen and Enli 2019), and IS (Mirbabaie et al. 2020).

### Digital Detox Research and Strategies

So far, empirical studies report mixed results concerning the effectiveness of digital detox (Wilcockson et al. 2019; Brown and Kuss 2020; Schmuck 2020), while also stating the importance of more research on this matter. The ambiguity of empirical findings, however, is partly due to an inconsistent and often vague conceptualization of ‘digital detox’. Moreover, viewed through a technostress lens, the phenomenon has been researched under the assumption of it being merely a coping strategy. This is reflected in recent definitions of digital detox, describing a process in which an individual abstains from objects that are perceived as unhealthy once exposure to them surpasses a certain point (Syvertsen and Enli 2019). Other terms like “digital diet” or “media diet” (Andersen et al. 2016) revolve around the same phenomenon as digital detox, which complicates consensus building. To establish the basis for a sound conceptualization of digital detox, Table 2 provides an overview of digital detox strategies that are prevalent in the literature.

**Table 2:** Examples of digital detox strategies covered by literature

Focus	Strategy	Supporting literature
Emotion-focused strategies (individual)	Reflecting personal values and mindset	Middleton and Cukier (2006); Syvertsen and Enli (2019); Pfaffinger et al. (2020)
	Emotion management	Al-Fudail and Mellar (2008)
	Mindfulness training	Pflügner et al. (2021)
Problem-focused strategies (individual)	Non-use, withdrawal, and time-outs	Baumer et al. (2013); Braukmann et al. (2018); Mirbabaie et al. (2020); Przybylski et al. (2021)
	Self-regulation of usage behavior	Uhls et al. (2014); Turel (2016); Anrijs et al. (2018); Stadin et al. (2020)
	Job transition	Butts et al. (2015)
	Restricted social media use	Gui et al. (2017); Aranda and Baig (2018); Karmakar (2020)
	Use of digital wellbeing applications	Gui et al. (2017); Eichner (2020); Karmakar (2020)
	Switching to alternatives and offline behaviors	D’Arcy et al. (2014); Syvertsen and Enli (2019)
	Segmentation of work and non-work	Sonnentag and Fritz (2015)
Problem-focused strategies (organizational)	Top-down regulation of usage behavior (e.g. shut down e-mail servers)	Görland and Kannengießer (2021)
	Organizational digital detox events	Karlsen (2020)
	Training and support	Tarafdar et al. (2011); Pfaffinger et al. (2020)

For the purpose of this article, we refer to digital detox as an integrated approach to temporarily refrain from IT use to improve overall well-being and mental health. In doing so, we want to emphasize the *preventive* element of digital detox in addition to the *coping* element, as proposed by the transactional model of technostress (Adam et al. 2017). Henceforth, this catchword paper aims to shed light on the different strategies of digital detox and to establish the concept as a proper subsumption to the technostress literature.

### Toward a Conceptualization of Digital Detox in Organizational Contexts

**Fig. 2:**
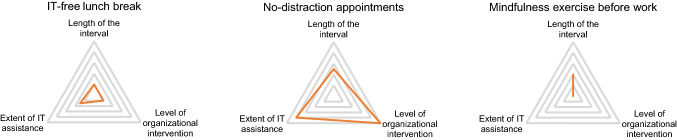
Examples of digital detox strategies and their specification

In the following, we aim to provide a first conceptualization of digital detox in organizations. As a first step, we take the identified digital detox strategies as shown in Table 1, and abstract three theoretical dimensions. First, we propose that the ‘length of the interval’ should be subject of scrutiny when researching digital detox. Second, we derive from existing strategies that they differ in the ‘extent of intervention’, that is in considering how much a digital detox strategy comes into conflict with organizational processes, norms, and behavior. Third, digital detox can have different ‘levels of IT-assistance’, e.g., through mindfulness apps, disabled e-mail servers after working hours, or calendar reminders to practice digital detox. Figure 2 provides three examples that range differently across the three dimensions of digital detox.

In this example, IT-free lunch breaks are minimal in length, intervention, and need for IT assistance. No-distraction appointments, on the other hand, can be medium in length and high in intervention and IT assistance. A mindfulness exercise may be chosen with short to medium length, no means of IT assistance and no interventions of organizational processes, norms, and behavior.

**Fig. 3:**
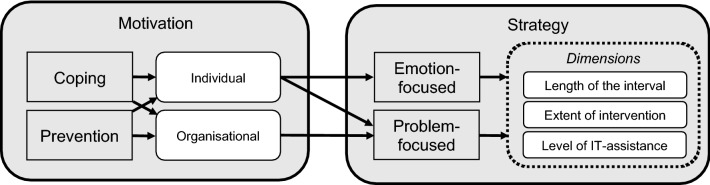
Conceptualization of digital detox in organizations

Below, we combine these dimensions with the transactional perspective known from the (organizational) technostress literature. Here, we consider both the motivational component of digital detox as well as the characteristics of a given digital detox strategy. Figure 3 depicts this integrated concept of digital detox.

This concept is useful for future research on digital detox for two reasons. First, digital detox proves to be a valuable concept to technostress and adjacent literature as it expands the theoretical chain of causation prevalent in current literature (stressors-strain-consequences *followed by* coping strategies to stressors-strain-consequences *preceded by* prevention strategies). Second, it allows for future research to determine a grading of digital detox strategies. This means that each measure (preventive or coping in nature) can be assessed and compared along the three dimensions.

## Research Agenda and Summary

In conclusion, the interdependencies of knowledge work arrangements and technostress make a strong case for more research exploring the phenomenon of digital detox. Moreover, additional theorizing is necessary to understand, explain, and predict behavior related to digital detox. In this regard, it is imperative for IS research to examine how digital detox strategies can prevent technostress and what value this perspective adds to existing coping strategies (see Fig. 1). Possible research questions are:

- What are individual motivators for knowledge workers to conduct digital detox?
- How do individual digital detox strategies differ when motivated by prevention as opposed to coping?
- Which techno-stressors can be mitigated by preventive digital detox strategies?

Methodical approaches to these types of questions can vary, however, qualitative-interpretivist inquiries will help to obtain a phenomenological angle to digital detox. It is crucial to understand how individuals experience digital detox, what motivates them, and how it changes their work processes and technology use.

In addition, subsequent questions emerge when shifting to an organizational view on digital detox. As proposed by the transactional model of technostress, factors such as job characteristics, technological environment, organizational environment, and the social environment impact the exposure to techno-stressors (Adam et al. 2017). This line of argumentation yields further research questions:

- How do job characteristics affect the conduct of digital detox?
- How is the technological environment (number and types of devices, software, etc.) interrelated with digital detox?
- To what extent is management responsible for ensuring an organizational environment that appreciates digital detox?
- What are the implications of digital detox for the social environment inside organizations, e.g., in times of social isolation?

Here, hypothetico-deductive methods using quantitative data will help to test the theoretical relationships of digital detox, e.g., building on the transactional model of technostress.

In existing studies, due to conceptual ambiguity, the effectiveness of digital detox has been assessed with mixed results. Therefore, it will be important to clearly define what digital detox strategies are and how their impact can be measured. We call for research that systematically creates a taxonomy for digital detox strategies, e.g., by considering the proposed dimensions ‘length of the interval’, ‘extent of intervention’, and ‘levels of IT-assistance’. Possible research questions in this regard are:

- What are digital detox strategies and how do they differ (e.g., from shortest and least interfering to longest and most interfering)?
- What are the implications of digital detox for IT design and the management of IT?

Finding answers to these questions will allow organizations, individual professionals, and IT designers to find a common ground that considers all interests at stake, that is, wellbeing, productivity, and user satisfaction.

In this catchword article, we proposed a first conceptualization of digital detox. We argue for digital detox as a phenomenon worthwhile to be examined as it adds a rather neglected perspective to technostress research. Instead of approaching technostress solely with coping mechanisms, digital detox offers a preventive and strategic element to technostress avoidance on both individual and organizational levels. We further stress the point that digital detox need not exclude IT dogmatically but should deploy it intelligently to support a user or organization to conscientiously implement digital detox strategies in their day-to-day operations. This will not only allow them to cope with technostress once a certain threshold is surpassed but helps to preventively regain equanimity and balance with regards to IT use. As Henry David Thoreau, the poet and philosopher we referenced in the opening quote to this article, would most certainly agree, digital detox may help us to elevate our lives by more conscious endeavors with and without IT.
